# A 7‐year prospective analysis of sustained benefits of multicomponent risk assessment and data‐driven care in patients with type 2 diabetes: The Malaysian JADE Program

**DOI:** 10.1111/dom.70125

**Published:** 2025-09-15

**Authors:** Jia‐Xin Hoo, Ya‐Feng Yang, Eric S. H. Lau, Luqman Ibrahim, Shireene R. Vethakkan, Jeyakantha Ratnasingam, Siew‐Pheng Chan, Sharmila S. Paramasivam, Yook‐Chin Chia, Alexander T. B. Tan, Andrea O. Y. Luk, Sanjay Rampal, Juliana C. N. Chan, Lee‐Ling Lim

**Affiliations:** ^1^ Department of Medicine, Faculty of Medicine Universiti Malaya Kuala Lumpur Malaysia; ^2^ Department of Medicine and Therapeutics The Chinese University of Hong Kong Hong Kong China; ^3^ Department of Medical Sciences Sunway University Petaling Jaya Malaysia; ^4^ Department of Primary Care Medicine, Faculty of Medicine Universiti Malaya Kuala Lumpur Malaysia; ^5^ Sunway Medical Centre Subang Jaya Malaysia; ^6^ Asia Diabetes Foundation Hong Kong China; ^7^ Hong Kong Institute of Diabetes and Obesity The Chinese University of Hong Kong, Prince of Wales Hospital Hong Kong China; ^8^ Li Ka Shing Institute of Health Sciences The Chinese University of Hong Kong, Prince of Wales Hospital Hong Kong China; ^9^ Department of Social and Preventive Medicine, Faculty of Medicine Universiti Malaya Kuala Lumpur Malaysia; ^10^ Baker Heart and Diabetes Institute Melbourne Victoria Australia

**Keywords:** data‐driven, diabetes, legacy effects, multicomponent care, technology, treatment targets

## Abstract

**Aim:**

We evaluated the sustained effects of multicomponent risk assessment and data‐driven care augmented by the web‐based Joint Asia Diabetes Evaluation (JADE) Platform with a built‐in template to guide comprehensive assessment (CA) for risk stratification on top of usual care in patients with type 2 diabetes (T2D) from Malaysia.

**Methods:**

In 2012–2015, 1196 T2D patients participated in a 1‐year JADE Program randomised to (1) CA‐only, (2) receipt of a JADE personalised report (CA + R) to empower self‐care, or (3) engagement by nurse phone calls (CA + R + T). In 2020–2022, patients underwent repeat CA for evaluation of attainment of ≥2 ABC targets (HbA1c <7%, Blood pressure <130/80 mmHg, low‐density lipoprotein Cholesterol [LDL‐C] <2.6 mmol/L) and diabetes‐related endpoints.

**Results:**

After 7.5 ± 0.5 (mean ± SD) years, 138 (11.5%) patients had died, 232 (19.4%) defaulted, and 826 (69.1%) patients returned. The deceased had more complications, while non‐returnees were younger, with fewer complications but worse risk factor control than returnees at baseline. Using inverse probability weighting and logistic regression, attaining ≥2 ABC targets was associated with CA + R + T (vs. CA‐only) with an odds ratio (OR, 95% confidence interval) of 1.57 (1.02–2.41, *p* = 0.041). Other predictors included age [1.05 (1.04–1.07, *p* < 0.001)], diabetes duration [0.97 (0.95–0.99, *p* = 0.003)], JADE risk category 3 [0.41 (0.26–0.66), *p* < 0.001] and risk category 4 (vs. category 1 and 2) [0.22 (0.12–0.38), *p* < 0.001]. Amongst participants without complications at baseline, CA + R + T was associated with an incidence rate ratio of 0.89 (0.78–1.00, *p* = 0.043) for any diabetes‐related endpoints.

**Conclusions:**

Technology‐assisted multicomponent risk assessment and data‐driven care for 1‐year identified high‐risk patients and improved outcomes after 7 years.

## 1 INTRODUCTION

In Malaysia, the prevalence of diabetes amongst adults increased from 11.2% in 2011 to 15.6% in 2023,^1^ with more than 99% of them having type 2 diabetes (T2D).^2^, ^3^ Patients with T2D have an increased risk for micro‐ and macrovascular complications^4^, ^5^, ^6^ which could be reduced by control of cardiometabolic risk factors.^7^ In the prospective Hong Kong Diabetes Register (HKDR), achieving ≥2 ABC targets (HbA1c <7%, Blood pressure [BP] <130/80 mmHg, or low‐density lipoprotein Cholesterol [LDL‐C] <2.6 mmol/L) was associated with a 30% reduced risk of cardiovascular disease (CVD) after a median follow‐up of 5.7 years.^8^ Suboptimal attainment of multiple treatment targets can lead to increased healthcare costs and poor quality of life.^9^, ^10^, ^11^ The heterogeneous and silent nature of T2D calls for quality improvement (QI) strategies targeting the healthcare system (e.g., relay of information), healthcare providers (e.g., task delegation) and patients (e.g., self‐management) to improve cardiometabolic risk factors.^12^, ^13^

The Joint Asia Diabetes Evaluation (JADE) Platform is a web‐based technology that incorporates guideline‐directed templates to guide regular comprehensive assessment (CA) (inclusive of eye/foot examination and blood/urine laboratory investigations) by allied health personnel and a risk engine including validated risk equations from the HKDR for risk stratification to enable users to establish a register for QI purposes. Using these comprehensive data, the JADE platform issues a personalised report (R) complete with JADE risk categories (JADE 1–4) based on different combinations of complications, risk factors and HKDR risk scores with trends, targets and decision support to empower self‐management and facilitate timely decision‐making. In 2012–2016, patients with T2D from Malaysia participated in a 1‐year multicomponent JADE Program on top of usual care.^14^ By reorganising the workflow and through task delegation, a doctor–nurse team implemented the JADE Program, where patients were randomised to either (1) CA (CA‐only); (2) CA with receipt of a personalised report (CA + R), or (3) CA with receipt of a personalised report and nurse telephone contacts (CA + R + T).^14^ In 2020–2022, we recalled these patients and assessed the sustained effects of the JADE Program and its validity in predicting clinical outcomes.

## 2 METHODS

### 2.1 Participants, setting, and JADE Program

This was a post‐trial evaluation study of patients with T2D from Malaysia who participated in the Asia‐Pacific JADE Program.^14^ Between 2012 and 2016, the Asia Diabetes Foundation (ADF), a non‐profit research organisation that designed, owned, and implemented the JADE Technology, conducted a 1‐year randomised QI program in eight Asian countries/regions including Malaysia. Patients with T2D aged ≥18 years treated with lifestyle modification and glucose‐lowering drugs, including insulin, were recruited into the Asia‐Pacific JADE Program.^14^ As part of this multicomponent JADE Program, nurses were trained to gather data using a case record form downloaded from the JADE Platform to guide CA, including laboratory investigations (blood/urine) and eye/foot examination.^13^ Data gathered included personal and medical history, vital signs, and anthropometric measurements. Sensory neuropathy was assessed by the 10‐g monofilament test and 128 Hz graduated tuning fork. Peripheral vascular disease (PVD) was detected by manual palpation of foot pulses or a Doppler scan. Eye examination included visual acuity assessment by the Snellen chart and ophthalmoscopy or fundus photography, read by doctors. Biochemical tests included full blood count, renal and liver function, fasting plasma glucose, HbA1c, fasting lipids, and urinary albumin: creatinine ratio (ACR). A 12‐lead electrocardiogram was also performed. This was followed by data entry, where patients were categorised into low risk (JADE risk category 1–2: no complication and few risk factors), high risk (JADE risk category 3: no history of CVD or end‐stage kidney disease [ESKD] but multiple risk factors including albuminuria [urinary ACR ≥3 mg/mmol], chronic kidney disease [CKD: estimated glomerular filtration rate [eGFR] <60 mL/min/1.73 m^2^] and/or high HKDR risk scores) and very high risk (JADE risk category 4: cardiovascular complications and/or ESKD, eGFR <15 mL/min/1.73 m^2^ or need for dialysis) with the issue of a personalised report.^15^

### 2.2 Malaysian JADE cohort

In 2012–2016, 1196 patients with T2D were recruited from Diabetes Specialist and Primary Care Clinics of the Universiti Malaya Medical Centre (UMMC). Eligible patients were randomised in a 2:1:1 ratio to (1) CA‐only without receipt of a personalised report; (2) CA + R with nurse explanation; and (3) CA + R + T with ≥2 telephone/face‐to‐face contacts by nurses over 1 year^14^ using computer‐generated codes placed in sealed, opaque envelopes opened by a personnel not involved in the randomised QI programme. Due to the pragmatic study design, group assignments were not blinded to patients, physicians, or nurses. All patients underwent CA after a 10‐h overnight fast at baseline and 12 months, on top of usual care.

In the present study, we recalled all surviving patients to undergo repeat JADE‐guided CA between 2020 and 2022. We ascertained clinical outcomes based on self‐reporting with verification from the UMMC electronic medical record (EMR) system and hospital discharge summaries from private and public healthcare settings, whenever appropriate. The study was conducted following the Declaration of Helsinki and approved by the UMMC Medical Research Ethics Committee (MREC ID: 20191216‐8082). All patients gave written informed consent before any study procedures.

### 2.3 Outcome definition

In this post‐trial evaluation analysis, the primary outcome was the attainment of ≥2 ABC targets (HbA1c <7%, Blood pressure [BP] <130/80 mmHg, and low‐density lipoprotein Cholesterol [LDL‐C] <2.6 mmol/L). We additionally evaluated a BP target <125/75 mmHg.^14^ The secondary outcome was the incidence of any diabetes‐related endpoints (retinopathy, peripheral sensory neuropathy, albuminuria, CKD, ischemic heart disease, stroke, peripheral vascular disease [PVD], or congestive heart failure). We used the National Death Registry to ascertain the death status censored on 28 February 2022. The first patient follow‐up visit was on 14 July 2020 and the last patient follow‐up visit was on 18 April 2022.

### 2.4 Statistical analysis

We presented descriptive data as mean ± standard deviation (SD), median (interquartile range, Q1–Q3), and number (percentage), whenever appropriate. We assessed data normality using the histograms, QQ plots, Shapiro–Wilk, and Kolmogorov–Smirnov tests. Data with skewed distributions were logarithmically transformed for analysis. For two‐group comparisons, we used either independent *t* test or Wilcoxon rank‐sum test for continuous variables with normal or skewed distributions, respectively. For three‐group comparisons, we used one‐way Analysis of Variance (ANOVA) and Kruskal–Wallis tests, as appropriate. For categorical variables, we used *χ* ^2^ or Fisher exact test for between‐group comparisons and the McNemar test for within‐group comparisons. We stratified the analyses by the status of follow‐up (returnees vs. non‐returnees) and survival (alive vs. deceased).

Due to attrition from death or default during the 7‐year period, there were differences in baseline profiles amongst the returnees randomised to the three groups. We applied inverse probability weighting (IPW) based on the present Malaysian subcohort's baseline characteristics to adjust for bias and confounding due to these differences. This was followed by logistic regression analysis to examine the effects of CA + R and CA + R + T versus CA‐only on the primary outcome, expressed as odds ratio (OR) with a 95% confidence interval (CI). In patients without complications at baseline, we compared the incidence rate ratio (with 95% CI) for diabetes‐related endpoints for interventions versus CA‐only. For the handling of missing data, we used listwise deletion for either logistic or linear regression analysis, and pairwise deletion for other descriptive analyses. All statistical analyses were conducted using the Statistical Package for Social Science (IBM, Version 28). A two‐sided *p*‐value <0.05 indicated statistical significance.

## 3 RESULTS

### 3.1 Clinical characteristics

**FIGURE 1:**
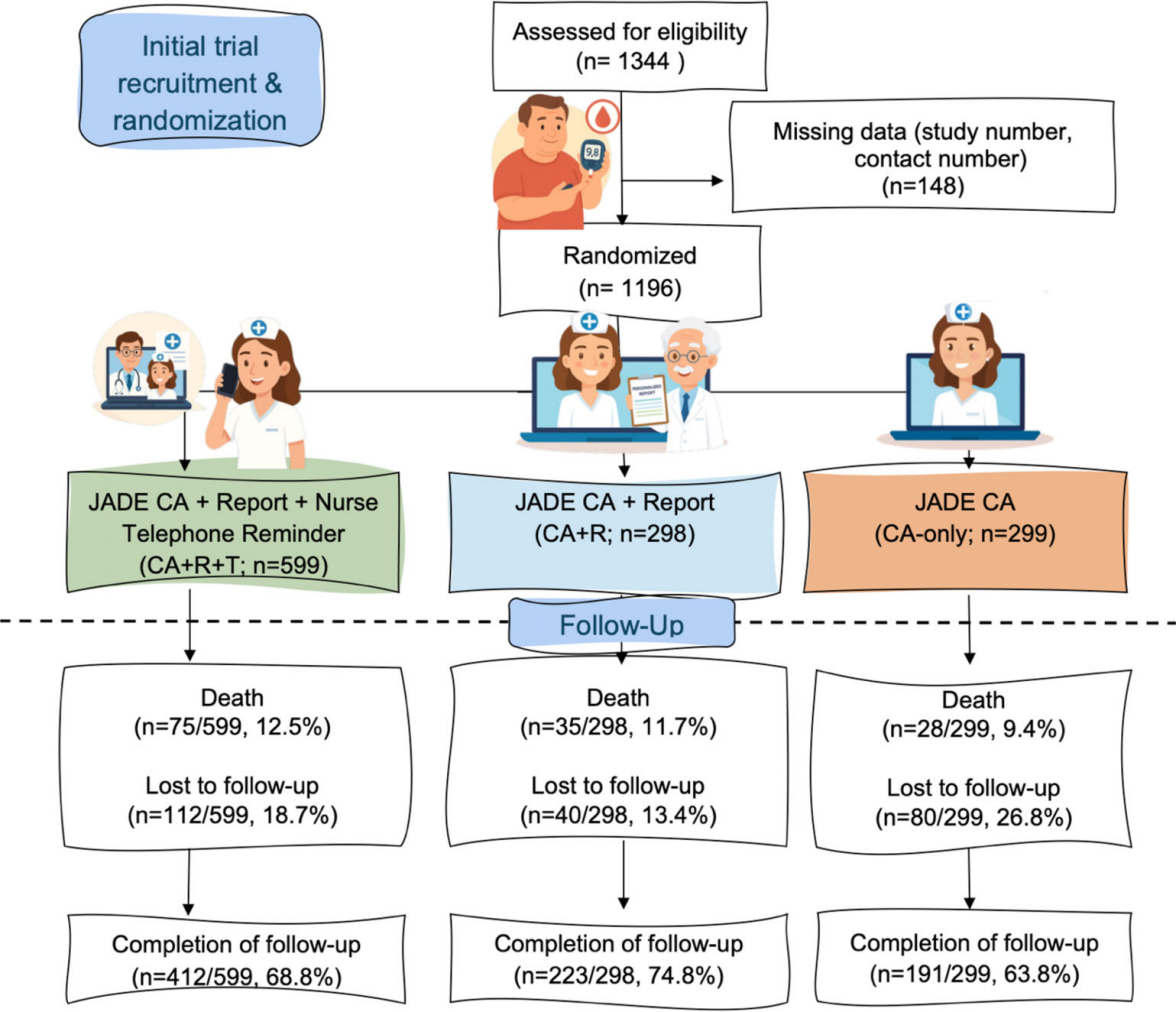
Study flow diagram for Malaysian subcohort in the Asia‐Pacific JADE Program (2012–2016) with recall at year 7 in 2020–2022. The reasons for lost‐to‐follow‐up were either patients' contact status not updated or patients' refusal to return due to fear of the COVID‐19 pandemic, emigration, or inability to return due to severe illnesses/injuries.

In 2012–2016, UMMC randomised 1196 patients with T2D, with 599 assigned to the CA + R + T group, 298 to the CA + R group, and 299 to the CA‐only group (Figure 1). At randomisation, the mean age was 60.1 ± 10.5 years, 616 (51.5%) were men, the median duration of diabetes was 10 (5.0–16.0) years, 35.6% achieved ≥2 ABC targets, 83.9% were on statin, and 61.5% were on renin‐angiotensin system inhibitors (RASi) (Table 1). By 2020–2022, 138 patients (11.5%) had died, 232 (19.4%) defaulted, and 826 (69.1%) returned for repeat CA (Figure 1). Amongst the deceased, 75 (12.5%) were from the CA + R + T group, 35 (11.7%) from the CA + R group, and 28 (9.4%) from the CA‐only group (Figure 1). The deceased were more likely to be men and older, with longer disease duration and lower education levels. They had worse cardiometabolic risk factors and complications, including CKD, retinopathy, CVD, and congestive heart failure, with a higher usage of insulin and RASi at baseline (Table S1). Compared with the returnees (*n* = 826), non‐returnees (*n* = 232) were younger and were more likely to work full time, with shorter disease duration, fewer complications, but worse lipid and glucose control at baseline. Self‐care behaviours, use of RASi, statin, insulin, and proportions of patients achieving ≥2 ABC targets were similar between returnees and non‐returnees (Table S2).

**TABLE 1:** Baseline characteristics of patients with type 2 diabetes (*n* = 1196) who participated in a 1‐year multicomponent quality improvement programme in 2012–2016, randomised to comprehensive assessment (CA‐only) guided by the Joint Asia Diabetes Evaluation (JADE) technology, CA followed by issue of a personalised report (CA + R) and CA + R with additional phone calls by nurses (CA + R + T).

Baseline characteristics	Total patients (*N* = 1196)	CA + R + T (*n* = 599)	CA + R (*n* = 298)	CA‐only (*n* = 299)
Sociodemographic				
Age, years	60.1 ± 10.5	59.9 ± 10.1	59.7 ± 10.9	61.0 ± 10.7
Duration of diabetes, median (Q1–Q3), years	10.0 (5.0–16.0)	10.0 (6.0–16.0)	12.0 (6.0–18.0)	8.0 (4.0–13.0)
Men, *n* (%)	616 (51.5%)	302 (50.4%)	161 (54.0%)	153 (51.2%)
College education or above, *n* (%)	373 (31.2%)	195 (32.8%)	96 (32.3%)	82 (27.6%)
Current smoker, *n* (%)	99 (8.3%)	49 (8.2%)	18 (6.1%)	32 (10.8%)
Ethnicity				
Indian, *n* (%)	582 (48.7%)	296 (50.9%)	150 (25.8%)	136 (23.4%)
Malays, *n* (%)	313 (26.2%)	151 (48.2%)	75 (24.0%)	87 (27.8%)
Chinese, *n* (%)	284 (23.7%)	144 (50.7%)	67 (23.6%)	73 (25.7%)
Others, *n* (%)	17 (1.4%)	8 (47.1%)	6 (35.3%)	3 (17.6%)
Job nature				
Retired, *n* (%)	660 (55.2%)	328 (49.7%)	170 (25.8%)	162 (24.5%)
Full time, *n* (%)	247 (20.7%)	124 (50.2%)	69 (27.9%)	54 (21.9%)
Others, *n* (%)	289 (24.2%)	147 (50.9%)	59 (20.4%)	83 (28.7%)
Self‐care practices				
Self‐monitoring of blood glucose, *n* (%)	789 (71.7%)	401 (71.1%)	224 (78.0%)	164 (65.6%)
Physical activity (3 times per week or more), *n* (%)	409 (34.6%)	187 (31.4%)	113 (37.9%)	109 (37.6%)
Balanced diet (yes or occasional), *n* (%)	877 (74.7%)	446 (75.6%)	224 (75.4%)	207 (72.1%)
Cardiometabolic risk factors				
FPG, median (Q1–Q3), mmol/L	7.3 (6.0–9.3)	7.4 (6.1–9.2)	7.3 (6.1–9.7)	7.1 (6.0–9.3)
HbA1c, mean ± SD, %	7.7 ± 1.8	7.8 ± 1.8	7.7 ± 1.6	7.8 ± 1.9
SBP, mean ± SD, mmHg	135.9 ± 16.9	135.9 ± 17.0	134.8 ± 16.0	137.0 ± 17.6
DBP, mean ± SD, mmHg	80.1 ± 9.9	80.2 ± 9.7	77.8 ± 10.2	82.2 ± 9.3
Total Cholesterol, mean ± SD, mmol/L	4.5 ± 1.1	4.5 ± 1.0	4.4 ± 1.0	4.6 ± 1.2
LDL‐C, mean ± SD, mmol/L	2.5 ± 0.9	2.5 ± 0.9	2.4 ± 0.8	2.7 ± 1.0
HDL‐C, median (Q1–Q3), mmol/L	1.1 (1.0–1.4)	1.1 (0.9–1.4)	1.2 (1.0–1.4)	1.2 (1.0–1.4)
Triglycerides, median (Q1–Q3), mmol/L	1.5 (1.1–2.1)	1.6 (1.1–2.2)	1.4 (1.0–1.9)	1.5 (1.1–2.1)
Waist circumference (men), mean ± SD, cm	97.0 ± 11.9	96.9 ± 11.5	97.7 ± 12.4	96.5 ± 12.5
Waist circumference (women), mean ± SD, cm	90.5 ± 10.9	91.0 ± 11.1	90.4 ± 11.6	89.5 ± 9.9
BMI, mean ± SD, kg/m^2^	27.8 ± 5.0	28.0 ± 5.0	28.3 ± 5.4	27.0 ± 4.8
Urinary ACR, median (Q1–Q3), mg/mmol	2.1 (0.7–8.6)	2.1 (0.7–8.4)	1.8 (0.7–6.9)	2.7 (1.1–12.2)
eGFR, mean ± SD, mL/min/1.73 m^2^	81.8 ± 22.6	82.1 ± 22.1	80.3 ± 25.2	82.8 ± 20.6
Frequency of self‐reported hypoglycemia ≥once/month, *n* (%)	133 (11.2%)	61 (10.2%)	38 (12.8%)	34 (11.4%)
Comorbidities, *n* (%)				
Chronic kidney disease	212 (17.7%)	103 (17.2%)	64 (21.5%)	45 (15.1%)
Sensory neuropathy	199 (16.7%)	99 (16.5%)	100 (33.6%)	0 (0%)
Diabetic retinopathy	75 (6.3%)	37 (6.2%)	37 (12.4%)	1 (0.3%)
Congestive heart failure	13 (1.1%)	8 (1.3%)	4 (1.3%)	1 (0.3%)
Cardiovascular disease	262 (21.9%)	142 (23.7%)	81 (27.2%)	39 (13.0%)
Ischemic heart disease	207 (17.3%)	107 (17.9%)	71 (23.8%)	29 (9.7%)
Stroke	54 (4.5%)	30 (5.0%)	14 (4.7%)	10 (3.3%)
Peripheral vascular disease	19 (1.6%)	16 (2.7%)	2 (0.7%)	1 (0.3%)
Any diabetes‐related complications	581 (48.6%)	294 (50.6%)	138 (51.3%)	149 (25.6%)
Medications, *n* (%)				
RASi	699 (61.5%)	350 (50.1%)	185 (26.5%)	164 (23.5%)
Statins	881 (83.9%)	459 (52.1%)	195 (22.1%)	227 (25.8%)
Oral glucose‐lowering drug	1146 (95.8%)	579 (50.5%)	281 (24.5%)	286 (25.0%)
Insulin	301 (25.2%)	161 (53.5%)	88 (29.2%)	52 (17.3%)
Treatment targets attainment, *n* (%)				
HbA1c <7.0%	469 (40.2%)	238 (50.7%)	109 (23.2%)	122 (26.0%)
BP <130/80 mmHg	248 (21.3%)	124 (50.0%)	85 (34.3%)	39 (15.7%)
LDL‐C <2.6 mmol/L	695 (60.8%)	354 (50.9%)	183 (26.3%)	158 (22.7%)
≥2 treatment targets attained	426 (35.6%)	210 (49.3%)	114 (26.8%)	102 (23.9%)
Frequency of calls, *n* (%)	Not applicable	561 (99.8%)	Not applicable	Not applicable
Non‐returnees at 7‐year, *n* (%)	232 (19.4%)	112 (18.7%)	40 (13.4%)	80 (26.8%)

*Note*: Data were indicated as mean ± standard deviation; *n* (%), number (percentage); or median (Q1–Q3). Cardiovascular disease included ischemic heart disease, peripheral vascular disease, and stroke.

Abbreviations: ACR, urine albumin/creatinine ratio; BMI, body mass index; BP, blood pressure; DBP, diastolic blood pressure; eGFR, estimated glomerular filtration rate; FPG, fasting plasma glucose; HbA1c, glycated haemoglobin; HDL‐C, high‐density lipoprotein cholesterol; LDL‐C, low‐density lipoprotein cholesterol; NGSP: National Glycohaemoglobin Standardization Program; RASi, Renin‐angiotensin‐aldosterone system inhibitors.

### 3.2 Primary outcome

Amongst the returnees, 348 (42.1%) patients achieved ≥2 ABC targets upon completion of the 1‐year randomised study (Table S3). After 7.5 ± 0.5 years, repeat CA indicated that 335 (40.6%) patients achieved ≥2 ABC targets and 491 (59.4%) did not. The former group was older and had better control of cardiometabolic risk factors at baseline. They were more likely to be Chinese and less likely to have prior CVD and to be treated with insulin (Table 2). Amongst patients who had data at baseline, year 1, and year 7, self‐care practices including regular physical activity and adherence to a balanced diet had improved at year 1, which was sustained at year 7 (Table S3). The proportions of patients with ≥2 ABC targets increased from 39.6% at baseline to 47.6% at year 1 and 39.3% at year 7 in the CA + R + T group (Figure S1A). The respective figures were 39.4%, 43.7%, and 41.7% in the CA + R group and 38.3%, 38.6%, and 39.8% in the CA‐only group (Figure S1A). Figure S1B–D showed the proportions of patients with individual ABC targets at baseline, year 1, and year 7, whilst Figure S2 displayed the trends in HbA1c, SBP, DBP, and LDL‐C across three timepoints.

**TABLE 2:** Trajectory and comparison of clinical characteristics of 826 returnees during the 1‐year randomised controlled trial of the JADE Programme (baseline and year 1) and post‐trial (year 7), stratified by attainment of 2 ABC targets.

	<2 ABC targets attained at year 7 (*n* = 491)				≥2 ABC targets attained at year 7 (*n* = 335)			
	Baseline	Year 1	Year 7	*p*‐value (ANOVA)	Baseline	Year 1	Year 7	*p*‐value (ANOVA)
Group assignment								
CA‐only	115 (23.4%)				76 (22.7%)			
CA + R	127 (25.9%)				96 (28.7%)			
CA + R + T	249 (50.7%)				163 (48.7%)			
Sociodemographic								
Age, years	58.9 ± 9.9	60.0 ± 9.9	66.5 ± 9.9	<0.001	62.0 ± 8.8	63.0 ± 8.8	69.5 ± 8.9	<0.001
Duration of diabetes, median (Q1–Q3), years	10.0 (5.0–16.0)	11.0 (6.0–18.0)	18.0 (13.0–24.0)	<0.001	9.0 (5.0–15.0)	10.0 (6.0–16.0)	16.0 (12.0–23.0)	<0.001
Men, *n* (%)	252 (51.3%)				172 (51.3%)			
College education or above, *n* (%)	161 (32.8%)				122 (36.4%)			
Current smoker, *n* (%)	29 (5.9%)	17 (3.5%)	20 (4.1%)	<0.001	20 (6.0%)	7 (5.0%)	20 (6.0%)	<0.001
Ethnicity, *n* (%)								
Indian	246 (50.2%)				167 (50.0%)			
Malays	135 (27.6%)				69 (20.7%)			
Chinese	100 (20.4%)				95 (28.4%)			
Others	9 (1.8%)				3 (0.9%)			
Self‐care, *n* (%)								
Self‐monitoring of blood glucose	337 (73.6%)	290 (59.1%)	233 (47.5%)	<0.001	218 (71.2%)	213 (63.6%)	164 (49.0%)	<0.001
Physical activity (≥3 times/week)	168 (34.2%)	158 (32.2%)	211 (43.0%)	0.172	124 (37.6%)	126 (37.6%)	178 (53.1%)	0.021
Balanced diet	361 (73.5%)	388 (79.0%)	398 (81.1%)	<0.001	252 (75.2%)	291 (86.9%)	290 (86.6%)	<0.001
Cardiometabolic risk factors								
FPG, median (Q1–Q3), mmol/L	7.6 (6.3–9.7)	7.5 (6.3–9.5)	8.0 (6.5–9.9)	0.048	6.8 (5.9–8.1)	6.8 (5.9–8.0)	6.4 (5.5–7.3)	0.007
HbA1c, mean ± SD, %	8.1 ± 1.7	7.7 ± 1.6	8.4 ± 1.8	<0.001	7.1 ± 1.3	6.9 ± 1.2	6.7 ± 1.0	<0.001
SBP, mean ± SD, mmHg	135.9 ± 16.4	133.7 ± 15.0	147.6 ± 16.1	<0.001	133.6 ± 16.9	130.1 ± 13.6	137.6 ± 19.6	<0.001
DBP, mean ± SD, mmHg	80.7 ± 10.1	77.3 ± 9.1	75.4 ± 8.9	<0.001	78.4 ± 9.3	75.5 ± 8.0	71.2 ± 10.0	<0.001
Total Cholesterol, mean ± SD, mmol/L	4.5 ± 1.1	4.4 ± 0.9	4.6 ± 1.3	0.007	4.3 ± 0.9	4.2 ± 0.8	3.9 ± 0.8	<0.001
LDL‐C, mean ± SD, mmol/L	2.5 ± 0.9	2.4 ± 0.8	2.6 ± 1.2	0.139	2.4 ± 0.9	2.2 ± 0.7	2.0 ± 0.6	<0.001
HDL‐C, median (Q1–Q3), mmol/L	1.2 (1.0, 1.4)	1.2 (1.0, 1.4)	1.2 (1.0, 1.4)	<0.001	1.2 (1.0, 1.4)	1.2 (1.0, 1.4)	1.2 (1.0, 1.5)	<0.001
Triglycerides, median (Q1–Q3), mmol/L	1.5 (1.1, 2.0)	1.3 (1.0, 1.8)	1.4 (1.1, 1.9)	<0.001	1.4 (1.1, 2.0)	1.3 (1.0, 1.8)	1.2 (0.9, 1.6)	<0.001
Waist circumference (men), mean ± SD, cm	97.8 ± 12.4	99.5 ± 11.7	97.6 ± 11.7	0.010	95.4 ± 11.8	96.9 ± 10.9	94.2 ± 11.6	<0.001
Waist circumference (women), mean ± SD, cm	91.5 ± 10.7	93.5 ± 10.9	94.4 ± 11.9	<0.001	89.0 ± 11.3	91.6 ± 10.0	88.9 ± 11.5	<0.001
BMI, mean ± SD, kg/m^2^	28.2 ± 4.8	28.2 ± 4.9	27.8 ± 5.2	<0.001	27.2 ± 4.8	27.0 ± 4.8	26.1 ± 5.2	<0.001
Urinary ACR, median (Q1–Q3), mg/mmol	2.1 (0.7–8.3)	2.2 (0.7–7.2)	4.7 (1.7–22.8)	<0.001	1.4 (0.6–4.6)	1.3 (0.6–4.4)	2.7 (1.3–6.7)	<0.001
eGFR, mean ± SD, mL/min/1.73 m^2^	83.4 ± 22.2	86.0 ± 22.3	79.5 ± 26.1	<0.001	82.5 ± 20.1	84.2 ± 21.4	77.8 ± 22.8	<0.001
Frequency of self‐reported hypoglycaemia ≥once/month, *n* (%)	53 (10.8%)	45 (9.2%)	53 (10.8%)	<0.001	39 (11.6%)	14 (4.2%)	25 (7.5%)	<0.001
Comorbidities, *n* (%)								
Chronic kidney disease	72 (14.7%)	61 (12.4%)	104 (21.2%)	<0.001	51 (15.2%)	48 (14.3%)	74 (22.1%)	<0.001
Sensory neuropathy	102 (20.8%)	71 (14.5%)	217 (44.2%)	0.016	57 (17.0%)	47 (14.0%)	141 (42.1%)	0.004
Diabetic retinopathy	32 (6.5%)	31 (6.3%)	69 (14.1%)	<0.001	15 (4.5%)	22 (6.6%)	17 (5.1%)	<0.001
Congestive heart failure	5 (1.0%)	47 (14.0%)	47 (14.0%)	<0.001	0 (0.0%)	0 (0.0%)	5 (1.5%)	<0.001
Cardiovascular disease	124 (25.3%)	118 (24.0%)	193 (39.3%)	<0.001	56 (16.7%)	57 (17.0%)	117 (34.9%)	<0.001
Ischemic heart disease	95 (19.3%)	94 (19.1%)	136 (27.7%)	<0.001	46 (13.7%)	47 (14.0%)	90 (26.9%)	<0.001
Stroke	28 (5.7%)	26 (5.3%)	50 (10.2%)	<0.001	10 (3.0%)	9 (2.7%)	23 (6.9%)	<0.001
Peripheral vascular disease	9 (1.8%)	8 (1.6%)	42 (8.6%)	<0.001	4 (1.2%)	3 (0.9%)	25 (7.5%)	<0.001
Any diabetes‐related endpoints	167 (34%)	165 (33.6%)	357 (52.3%)	0.299	100 (29.7%)	102 (30.3%)	172 (51.0%)	0.623
Medications, *n* (%)								
RASi	193 (39.3%)	174 (35.4%)	190 (38.7%)	<0.001	124 (37.0%)	117 (34.9%)	110 (32.8%)	<0.001
Statins	363 (73.9%)	357 (72.7%)	368 (74.9%)	<0.001	257 (76.7%)	255 (76.1%)	270 (80.6%)	<0.001
Oral glucose‐lowering drugs	474 (96.5%)	433 (88.2%)	459 (93.5%)	<0.001	325 (97.0%)	310 (92.5%)	317 (94.6%)	<0.001
Insulin	144 (29.3%)	131 (26.7%)	245 (49.9%)	0.964	46 (13.7%)	45 (13.4%)	89 (26.6%)	<0.001

*Note*: Data are reported as mean ± standard deviation; *n* (%), number (percentage); or median (Q1–Q3). Cardiovascular disease includes coronary heart disease, stroke and peripheral vascular disease.

Abbreviations: ACR, albumin: creatinine ratio; BMI, body mass index; BP, blood pressure; DBP, diastolic blood pressure; eGFR, estimated glomerular filtration rate (in creatinine‐based CKD‐EPI formula); FPG, fasting plasma glucose; HbA1c, glycated haemoglobin; HDL‐C, high‐density lipoprotein cholesterol; LDL‐C, low‐density lipoprotein cholesterol; NGSP, National Glycohaemoglobin Standardization Program; RASi, renin‐angiotensin‐aldosterone system inhibitors.

**FIGURE 2:**
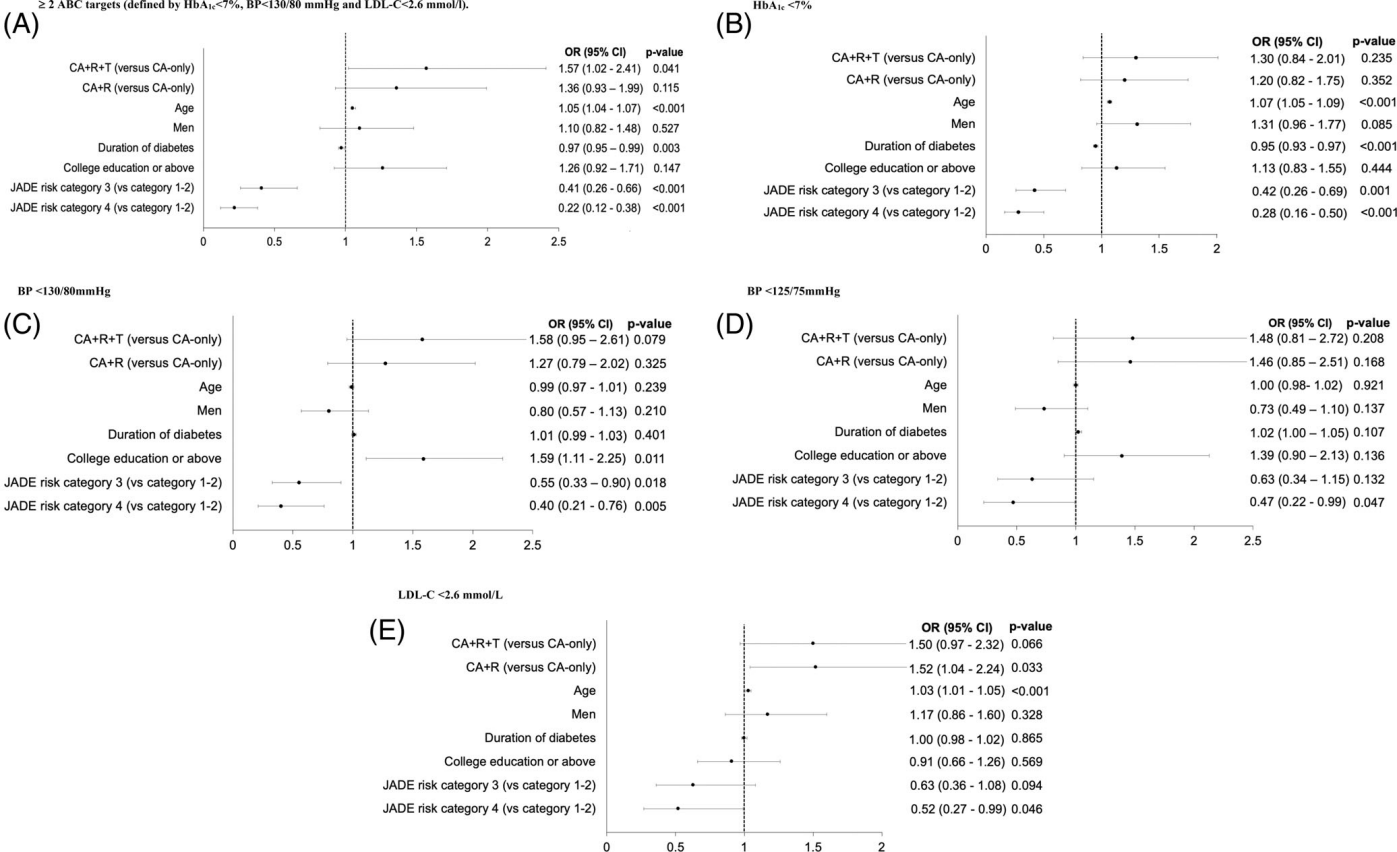
Forest plots showing the odds ratio with 95% confidence interval associated with attainment of ≥2 ABC targets and its various components at year 7 in 2020–2022 after exposure to 1 year of multicomponent JADE Program with assignment to comprehensive assessment (CA) only, CA + personalised report (CA + R) and CA + R + telephone contact by nurses (CA + R + T) using the inverse probability weighting method to adjust for baseline differences and multiple logistic regression analysis. OR (95% CI), odds ratio (95% confidence interval).

We applied IPW and logistic regression, and used CA‐only (*n* = 191) as the reference group. Attaining ≥2 ABC targets was associated with CA + R + T intervention with an OR of 1.57 (95% CI: 1.02–2.41, *p* = 0.041), age [1.05 (1.04–1.07), *p* < 0.001], diabetes duration [0.97 (0.95–0.99 *p* = 0.003)], JADE risk category 3 [0.41 (0.26–0.66), *p* < 0.001] and risk category 4 (vs. category 1 and 2) [0.22 (0.12–0.38), *p* < 0.001] (Figure 2).

### 3.3 Secondary outcome

After excluding patients with a prior history of complications, 573 patients had 359 incidents of any diabetes‐related endpoints during the 7.5‐year post‐trial evaluation period. In a multivariable model, JADE risk categories 3 and 4, older age, and longer disease duration were independently associated with any diabetes‐related endpoints (Table S4). Amongst patients without complications at baseline, IPW and logistic regression indicated that CA + R + T was associated with an incidence ratio of 0.89 (0.78–1.00, *p* = 0.043) for any diabetes‐related endpoints (Table 3).

**TABLE 3:** Using the inverse probability weighting method to adjust for baseline differences and multiple logistic regression analysis to compare the incidence rate ratio of all diabetes‐related endpoints at year 7 amongst returnees without prior complications, stratified by group assignment at randomisation versus CA‐only.

	Incidence rate ratio (95% CI)			
	CA + R + T (vs. CA‐only)	*p*‐value	CA + R (vs. CA‐only)	*p*‐value
Any diabetes‐related endpoints (either macro‐ or microvascular disease)	**0.89 (0.78–1.00)**	**0.043**	0.92 (0.82–1.03)	0.138
A composite of microvascular disease	**0.83 (0.70–0.98)**	**0.027**	0.89 (0.77–1.03)	0.121
Sensory neuropathy	0.95 (0.74–1.22)	0.687	0.95 (0.76–1.19)	0.661
Diabetic retinopathy	**0.46 (0.26–0.83)**	**0.010**	0.75 (0.48–1.17)	0.200
Chronic kidney disease	0.86 (0.63–1.17)	0.338	1.00 (0.76–1.32)	0.996
A composite of macrovascular disease	1.07 (0.87–1.32)	0.517	1.00 (0.82–1.22)	0.976
Congestive heart failure	1.48 (0.38–5.68)	0.571	0.94 (0.26–3.48)	0.929
Cardiovascular disease	1.07 (0.86–1.31)	0.556	1.01 (0.83–1.23)	0.916
Ischemic heart disease	1.10 (0.86–1.41)	0.457	1.13 (0.88–1.44)	0.330
Stroke	1.10 (0.69–1.75)	0.703	0.88 (0.59–1.34)	0.558
Peripheral vascular disease	1.72 (0.85–3.46)	0.131	1.28 (0.66–2.50)	0.469

*Note*: JADE CA‐only was the referent group for incidence rate ratio analysis. Bold values indicate statistically significant results (*p* < 0.05).

Abbreviations: CA, comprehensive assessment; CI, confidence interval.

## 4 DISCUSSION

In this post‐trial evaluation analysis of the Malaysian JADE sub‐cohort, after a mean post‐trial period of 7.5 years and after adjusting for confounding due to attrition, patients who received a JADE personalised report and telephone calls by nurses (CA + R + T) were 57% more likely to achieve ≥2 ABC targets than patients who only underwent CA without reporting and feedback. Amongst patients without complications at baseline, the CA + R + T group was also 11% less likely to develop any diabetes‐related endpoints than the CA‐only group. In addition to age and disease duration, the JADE risk categories 3–4 based on various combinations of risk factors, complications and HKDR risk scores, also identified patients at high risk of poor control of risk factors and developing incident diabetes endpoints.

In the Asia‐Pacific JADE Program (2012–2016), which recruited 20,834 patients with T2D from eight countries including Malaysia, there were two phases where the CA + R + T group was compared with the CA + R (phase 1) or CA‐only (phase 2) group. Investigators from Malaysia participated in both phases; thus, the ratio of 2:1:1 (CA‐only, CA + R, and CA + R + T) in this post‐trial evaluation analysis.^14^ In the entire Asia‐Pacific JADE cohort, compared with CA‐only or CA + R, the CA + R + T group was 25%–34% more likely to achieve ≥2 ABC treatment targets on top of usual care.^14^, ^16^ After completion of the 1‐year Asia‐Pacific JADE Program,^14^ all patients continued to receive usual care with variations in quality of care and random chances for treatment optimisation.^17^

After 7 years, in Malaysia, we successfully recalled ~80% of surviving patients to undergo repeat JADE‐CA. Among these returnees, CA + R + T intervention was associated with 57% increased odds of sustained attainment of ≥2 ABC targets after adjusting for confounders. In the original randomised controlled trial (RCT), we also found that the issue of a personalised JADE report with or without nurse calls improved risk factor control in part due to improved risk awareness, self‐management, medication adherence, and treatment intensification.^16^, ^18^ During these 7 years, UMMC had adopted components of the JADE Program into routine care including structured evaluation, education, and empowerment, which might contribute to the sustained improvement in risk factor control. Although fewer non‐returnees had cardiovascular disease at baseline, they were more likely to have poor glucose and lipid control and less likely to be treated with statins. Given the higher number of defaulters in the CA‐only group, it is possible these patients might have more adverse outcomes. In support of the sustained benefits of CA + R + T intervention on the attainment of treatment targets, patients without complications at baseline were 11% less likely to develop any diabetes‐related endpoints compared with the CA‐only group.

In another 1‐year JADE‐based RCT which compared the additional effects of peer support on control of risk factors and patient‐reported outcomes, receipt of a personalised JADE report improved negative emotions, self‐management, empowerment, self‐efficacy, and treatment adherence.^18^ In this 7‐year post‐trial evaluation analysis, the improvement in physical activity and adherence to a balanced diet at year 1 was also sustained at year 7. Taken together, these findings support the utility of the JADE Platform in guiding CA for risk stratification with the issue of a personalised report to provide feedback and motivate behavioural change.

Despite these encouraging results, young patients and those with short disease duration or absence of clinically evident complications were less likely to return for repeat CA. Given the chronicity of diabetes and importance of early intervention,^19^, ^20^ additional efforts are needed to engage these seemingly low‐risk patients to undergo regular assessment to avoid silent deterioration of risk factors and development of complications.^21^ Diabetes is heterogeneous in its causes, trajectories, and consequences, with many unmet needs. To address these challenges, team‐based data‐driven integrated care with regular assessment will help stratify risk, empower self‐management, reduce therapeutic inertia, personalise care, achieve control of risk factors early, and reduce long‐term complications.^13^ The JADE Program is the first web‐based platform aimed at enabling the care team to implement a multicomponent QI program integrating evaluation, empowerment, and engagement with the establishment of a register for benchmarking and QI purposes.^13^

Since its launching in 2007, more than 120 000 patients from 8 countries in Asia have been exposed to the JADE Program.^22^, ^23^ While EMR has improved the efficiency of data gathering, analytics, and reporting, a well‐executed structured assessment to guide personalised feedback and decision support has offered additional engagement and benefits. In a public–private partnership setting, JADE‐guided risk assessment and data‐driven care have reduced major events including death by 23%–36%, compared with comprehensive assessment without other components of risk stratification, reporting, and feedback.^24^ Results from this 7‐year follow‐up analysis of the JADE‐Malaysia study further support team‐based data‐driven care as a sustainable solution for improving clinical outcomes in patients with T2D who had complex phenotypes and multiple needs.^12^ The United Kingdom Prospective Diabetes Study (UKPDS) and Steno‐2 study have indicated that early control of cardiometabolic risk factors had legacy effects in reducing long‐term cardiovascular‐renal events and all‐cause death.^25^, ^26^, ^27^ In the Action in Diabetes and Vascular Disease: Preterax and Diamicron Modified Release Controlled Evaluation Observational (ADVANCE‐ON) Study, which recruited high‐risk patients, there was no difference in cardiovascular events between the intensive glycaemic control and usual care group after 11 years of follow‐up, although the predefined composite endpoint was positive in favour of intensive care.^28^ In the UKPDS, which recruited newly diagnosed patients, the legacy effects of improved glycaemic control on myocardial infarction and death were observed 10 years after completion of the 7‐year intervention.^29^ In this post‐trial analysis, after adjusting for confounding, the CA + R + T group was more likely to attain ≥2 ABC targets with lower complication rates amongst those without complications at baseline.

The importance of cardiovascular‐kidney‐metabolic (CKM) condition is recognised by cardiologists, diabetologists/endocrinologists, and nephrologists.^7^, ^30^, ^31^ The numerical, silent and progressive nature of these CKM conditions and complications calls for regular structured measurement for monitoring and management. The JADE risk categorisation encompasses modifiable and non‐modifiable risk factors, and together with the validated risk scores from the HKDR, facilitates the prediction and prognostication of outcomes. Due to the low number of events, we could not evaluate the performance of the HKDR risk scores, although the JADE risk categories predicted adverse outcomes in this multi‐ethnic cohort.^15^ Similar technologies^32^ are being developed to gather data or extract EMR data to drive actions, with the JADE Technology having the most implementation evidence.

In Hong Kong, the HKDR protocol developed in 1995 (the basis of the JADE protocol) was embedded in the territory‐wide EMR system since 2000 to provide a territory‐wide Risk Assessment and Management Program (RAMP), which has benefitted over 0.5 million people with diabetes. Despite variable fidelity in executing this multicomponent QI program in different care settings, analysis of the territory‐wide EMR system indicated 50%–70% risk reduction in major diabetes complications including all‐cause death in 2000–2016.^13^, ^33^ In a systematic analysis, amongst 16 high‐income jurisdictions with territory‐ or nationwide databases, Hong Kong had the largest decrement in diabetes‐related annual death rates from 3% in 2000 to 1.3% in 2016.^34^ The evaluation of this prospective Malaysian JADE subcohort provides further support to advocate payors and policymakers to invest and adopt this multicomponent risk assessment and data‐driven care model to improve outcomes, as exemplified by the JADE Program.

We acknowledge several limitations. Due to the pragmatic design, the study personnel were not blinded to the group assignment during the original Asia‐Pacific JADE RCT (2012‐2016). Amongst the survivors, only ~80% consented to return for follow‐up. This was considered reasonable given that the survey was conducted during the COVID‐19 pandemic. Volunteer bias might limit the generalisability of our findings and underestimate the true effect of data‐driven multicomponent integrated care. Similar to the cross‐sectional TARGET‐T2D study involving publicly funded hospitals in Malaysia,^35^ the present cohort had relatively good control of risk factors with a mean HbA1c of 8%, BP of 135/80 mmHg and LDL‐C of 2.5 mmol/L at baseline, which might reduce the impact of this care model. Fidelity to protocol implementation might affect the effects of the intervention, although we did not document details of various care components. Lifestyle changes during the COVID‐19 pandemic might or might not affect control of cardiometabolic risk factors.^36^, ^37^ In this post‐trial evaluation study, amongst the returnees, the improvement in self‐management at year 1 persisted into year 7. The patients were not systematically followed up during these 7 years. Since the onset of complications was not captured, we used logistic regression to evaluate the outcome, defined as a binary event at year 7 without accounting for varying follow‐up or censoring.

In conclusion, after a mean post‐trial evaluation period of 7.5 years, data‐driven multicomponent integrated care implemented through the JADE Platform led to sustained attainment of multiple treatment targets and reduced diabetes endpoints in patients with T2D. These long‐term data call for strengthening the healthcare system to implement a regular risk assessment and management programme plus personalised reporting for identifying high‐risk patients and improving clinical outcomes.

## AUTHOR CONTRIBUTIONS

Lee‐Ling Lim and conceptualised and designed the post‐trial evaluation study. Jia‐Xin Hoo, Ya‐Feng Yang, Eric S. H. Lau, Luqman Ibrahim, Shireene R. Vethakkan, Jeyakantha Ratnasingam, Siew‐Pheng Chan, Sharmila S. Paramasivam, Yook‐Chin Chia, Alexander T. B. Tan, Andrea O. Y. Luk, Sanjay Rampal, Lee‐Ling Lim, and Juliana C. N. Chan contributed to patient follow‐up and data acquisition. Jia‐Xin Hoo and Ya‐Feng Yang conducted the data analysis under the supervision of Lee‐Ling Lim, Eric S. H. Lau, and Juliana C. N. Chan. Jia‐Xin Hoo and Ya‐Feng Yang wrote the first draft. Lee‐Ling Lim and Juliana C. N. Chan finalised the manuscript. All authors contributed to data interpretation and critical revision of the manuscript. All authors approved the final version of the manuscript for submission.

## CONFLICT OF INTEREST STATEMENT

Luqman Ibrahim reported educational grants from Abbott, AstraZeneca, Boehringer Ingelheim, Ethos Healthcare, Medtronic, Novo Nordisk, Sandoz, and Zuellig Pharma; speaker honoraria from Abbott, AstraZeneca, Ethos Healthcare, Novo Nordisk, Pfizer, Sanofi, Viatris, and Zuellig Pharma. Jeyakantha Ratnasingam reported receiving grants and/or honoraria for consultancy or giving lectures from Abbott, Accord Pharma, Amgen, AstraZeneca, Bayer, Biomed Global, Boehringer Ingelheim, Ipsen, Novo Nordisk, Servier, Viatris, and Zuellig Pharma. Sharmila S. Paramasivam reported receiving honoraria for consultancy or giving lectures from AstraZeneca, Boehringer Ingelheim, Novo Nordisk, Brego, Viatris, and Zuellig Pharma. Siew‐Pheng Chan reported receiving grants and/or honoraria for consultancy or giving lectures from Abbott, AstraZeneca, Bayer, Merck Serono, Novo Nordisk, Sanofi, Servier, Viatris, and Zuellig Pharma. Andrea O. Y. Luk reported advisory panel: Amgen, AstraZeneca, Boehringer Ingelheim, and Sanofi; research support/travelling grant: Amgen, Asia Diabetes Foundation, AstraZeneca, Bayer, Biogen, Boehringer Ingelheim, Junshi, Lee's Pharmaceutical, MSD, Novo Nordisk, Roche, Sanofi, Sugardown Ltd., and Takeda. Juliana C. N. Chan reported receiving research grants through her affiliated institutions, honorarium, and speakers' fees from Abbott, Applied Therapeutics, AstraZeneca, Bayer, Boehringer Ingelheim, Celltrion, Hua Medicine, Lee Powder, Lilly, Merck Sharpe Dohme, Merck Serono, Pfizer, Roche, Sanofi, Servier, Viatris, and Zuellig Pharma. She holds patents awarded to the Chinese University of Hong Kong for using genetic markers to predict diabetes and its complications for personalised care. She is a co‐founder of a biotechnology start‐up company, GemVCare, with partial support from the Hong Kong Government Innovation and Technology Commission for providing precision diabetes care and Chief Executive Officer (pro bono) of Asia Diabetes Foundation, which developed the JADE Platform. Lee‐Ling Lim reported receiving grants and/or honoraria for consultancy or giving lectures from Abbott, AstraZeneca, Bayer, Boehringer Ingelheim, Novartis, Novo Nordisk, Roche, Viatris, and Zuellig Pharma. Other authors declare no potential conflict of interest.

## Supporting information


**Data S1.** Supporting Information.

## Data Availability

Data available in article supplementary material.
