# Supplementary material for: A 7‐year prospective analysis of sustained benefits of multicomponent risk assessment and data‐driven care in patients with type 2 diabetes: The Malaysian JADE Program
Source: Diabetes Obes Metab. 2025 Sep 15;27(12):7232–43. doi: 10.1111/dom.70125 (PMC12587254; doi:10.1111/dom.70125)
Supplement: Supplementary file 1 — Data S1. Supporting Information. [file DOM-27-7232-s001.pdf]

Supplementary Materials

A 7-year prospective analysis of sustained benefits of multicomponent risk assessment and data-driven care in patients with type 2 diabetes: the Malaysian JADE Program

Contents

Figure S1. Proportions of patients with (A) ≥2 ABC targets, (B) HbA_{1c}<7%, (C) BP<130/80 mmHg, (D) LDL-C<2.6 mmol/L during the 1-year randomized controlled trial of the JADE Program (baseline and year 1) and post-trial (year 7)..2

Figure S2. Change of (A) HbA_{1c}, (B) SBP, (C) DBP, (D) LDL-C from baseline to year 1 and post-trial (year 7) 6

S1 Table. Baseline characteristics of patients with type 2 diabetes confirmed to be alive or deceased at year 7...8

S2 Table. Comparison of baseline characteristics between patients who did or did not return for follow-up 10

S3 Table. Comparison of clinical characteristics of 826 returnees during the 1-year randomized controlled trial of the JADE Program (baseline and year 1) and post-trial (year 7) 12

S4 Table. Logistic regression analysis of factors associated with any incident diabetes-related endpoints amongst patients without prior history of complications.........................14

Figure S1. Proportions of patients with (A) ≥2 ABC targets, (B) HbA_{1c}<7%, (C) BP<130/80 mmHg, (D) LDL-C<2.6 mmol/L during the 1-year randomized controlled trial of the JADE Program (baseline and year 1) and post-trial (year 7)

(A) ≥2 ABC targets

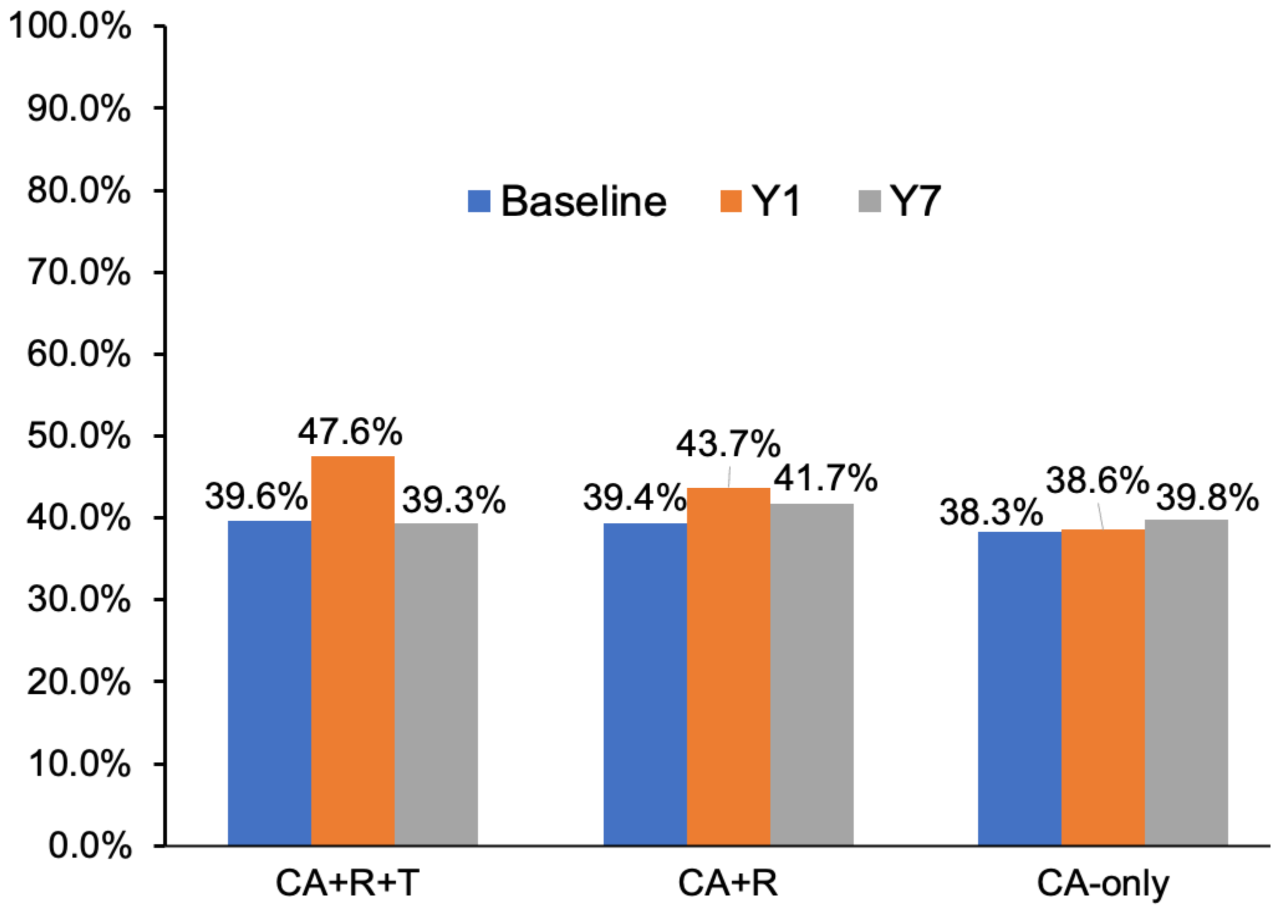

(B) HbA_{1c}<7%

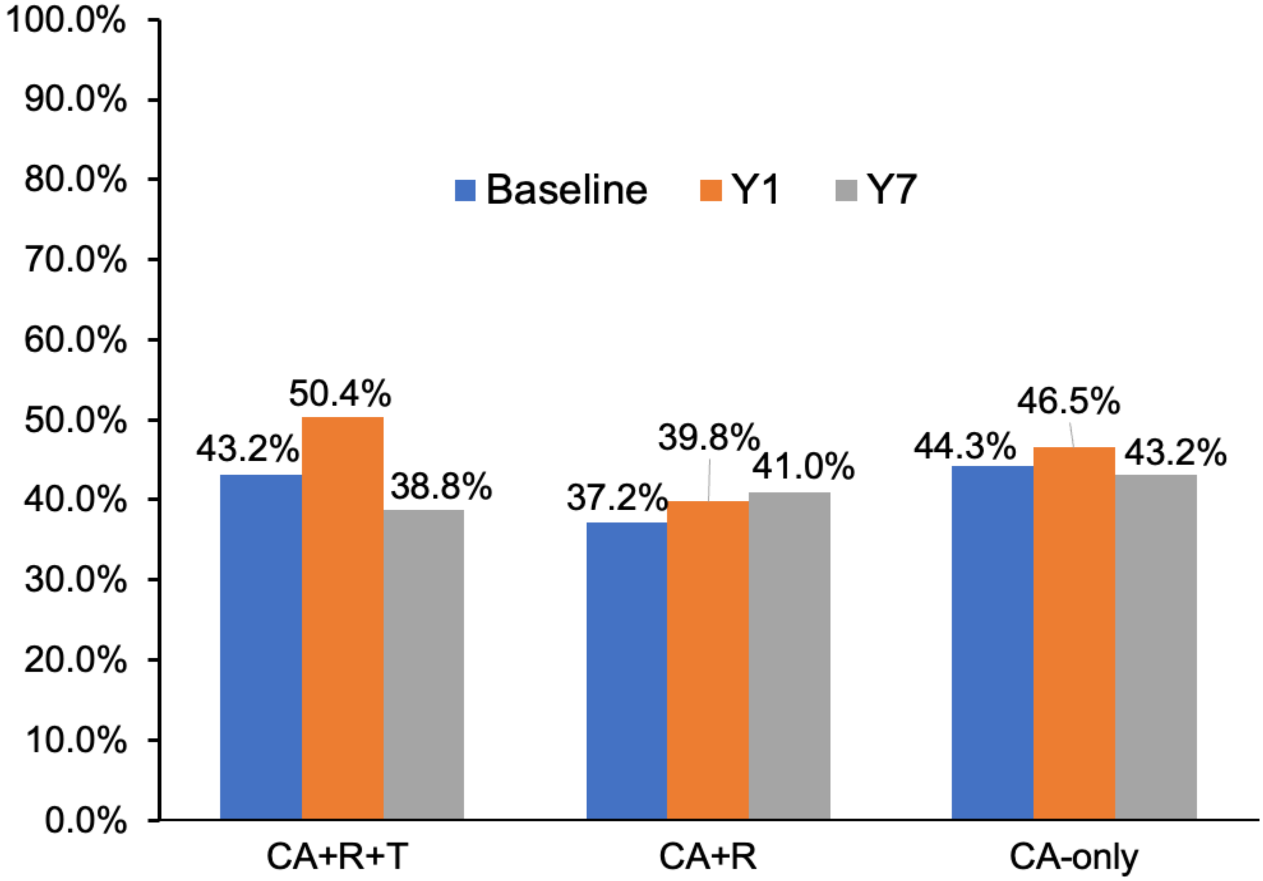

100.0%
90.0%
80.0%
70.0%
60.0%
50.0%
40.0%
30.0%
20.0%
10.0%
0.0%
Baseline
Y1
Y7
43.2%
50.4%
38.8%
37.2%
39.8%
41.0%
44.3%
46.5%
43.2%
CA+R+T
CA+R
CA-only

(C) BP<130/80 mmHg

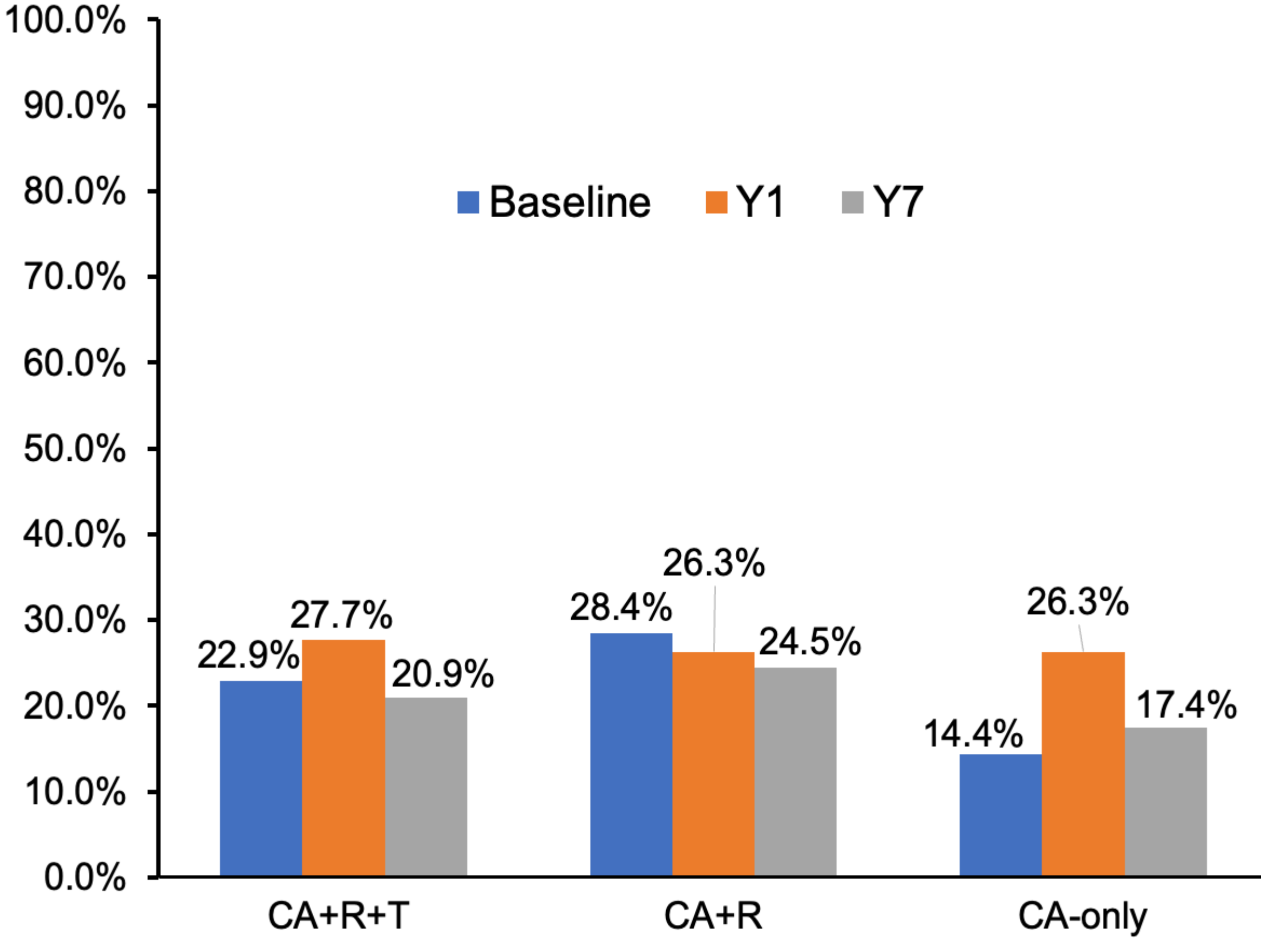

(D) LDL-C<2.6 mmol/L

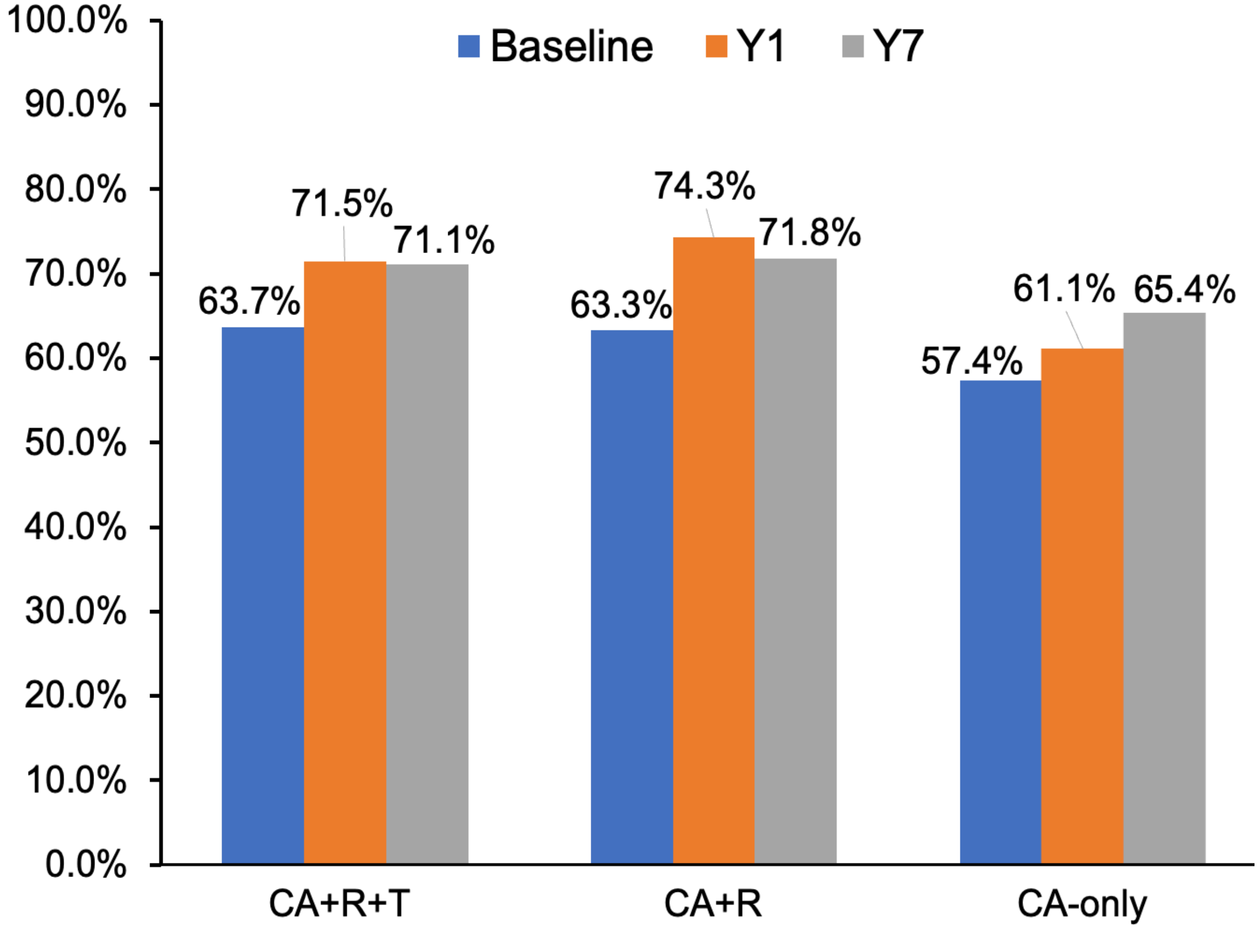

Footnotes: CA+R, CA+personalized report; CA+R+T, CA+R+telephone contact by nurses. CA, comprehensive assessment.

Figure S2. Change of (A) HbA_{1c}, (B) SBP, (C) DBP, (D) LDL-C from baseline to year 1 and post-trial (year 7)

(A) HbA_{1c}

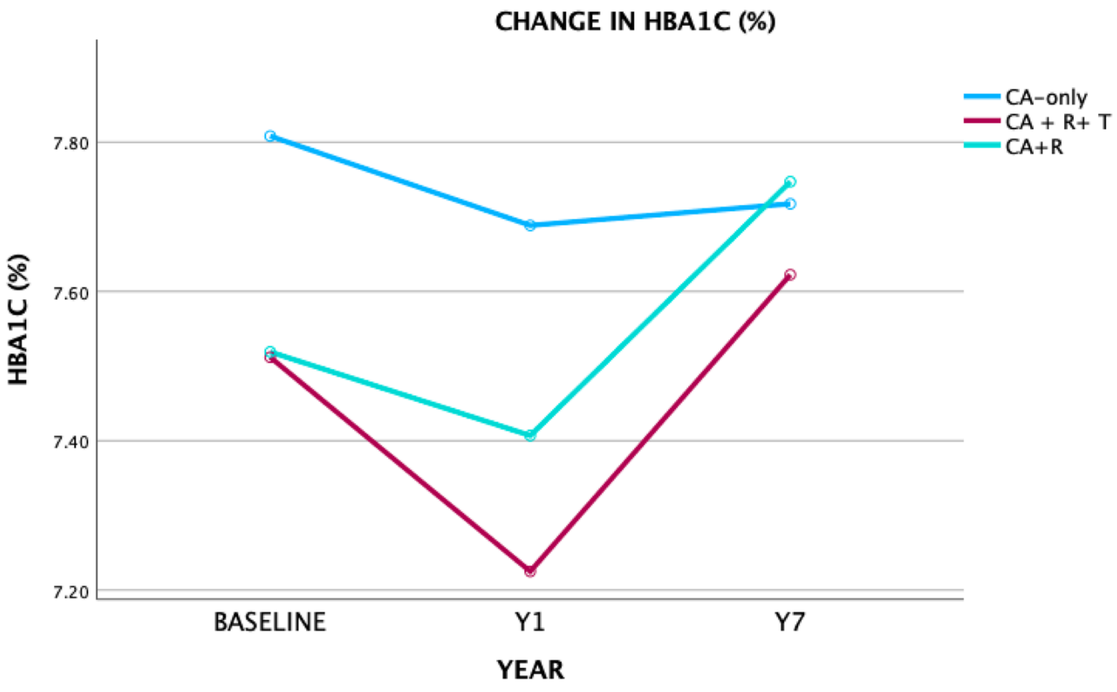

(B) SBP

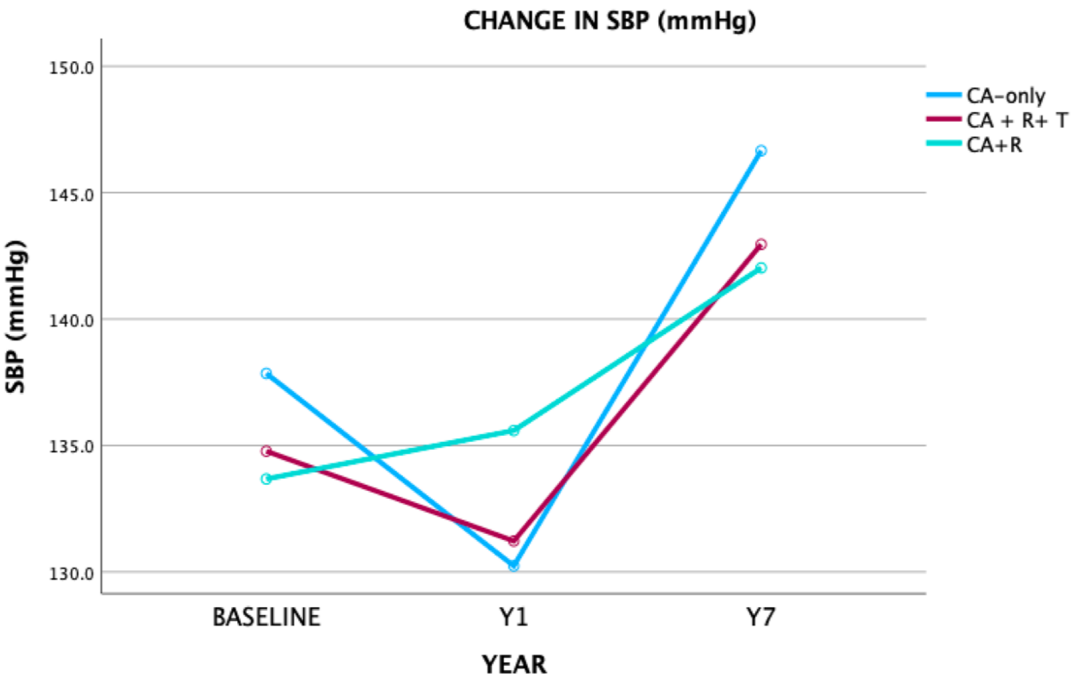

(C) DBP

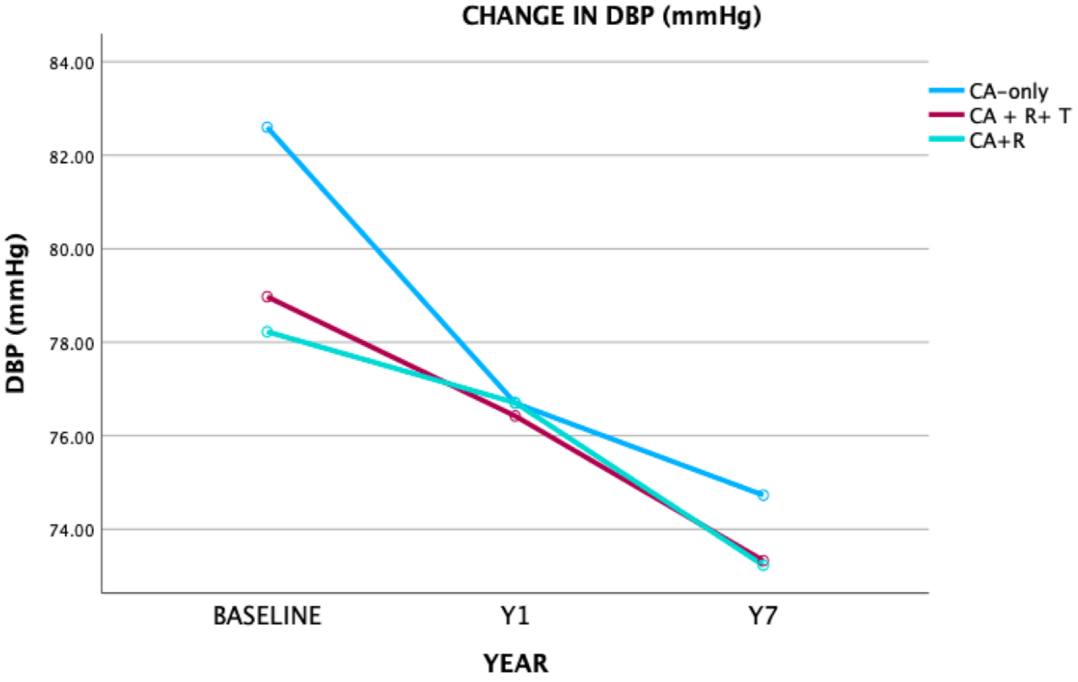

(D) LDL-C

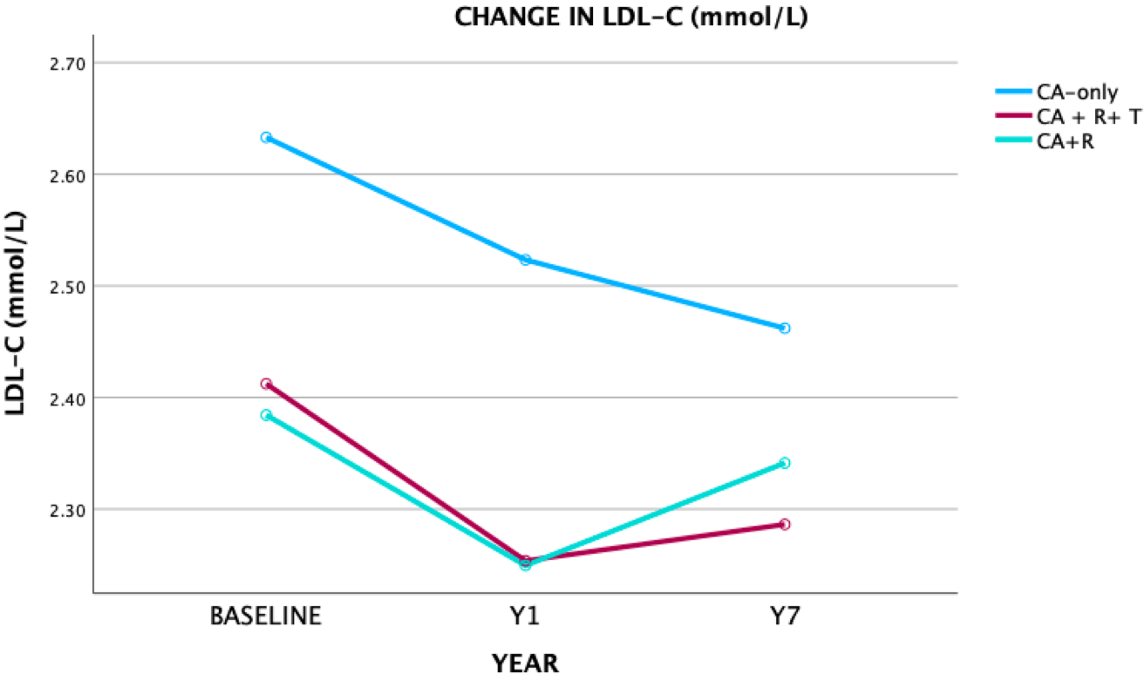

Footnotes: All models were adjusted with age, male gender, duration of diabetes, college education or above, and JADE risk categories; HbA_{1c}, glycated hemoglobin; LDL-C, low-density lipoprotein cholesterol.

S1 Table. Baseline characteristics of patients with type 2 diabetes confirmed to be alive or deceased at year 7.

Characteristic	n	Alive (n=1058)	n	Deceased (n=138)	p-value
**Assigned Intervention**					
CA-only	1058	271 (25.6%)	138	28 (20.3%)	0.375
CA+R		263 (24.9%)		35 (25.4%)	
CA+R+T		524 (49.5%)		75 (54.3%)	
**Sociodemographic**					
Age, years	1058	59.5 ± 10.3	138	65.1 ± 10.2	<0.001
Duration of diabetes, median (Q1-Q3), years	1054	9.0 (5.0-15.0)	136	13.5 (9.0-20.0)	<0.001
Men, n (%)	1058	529 (50.0%)	138	87 (63.5%)	0.004
College education or above, n (%)	1056	359 (34.0%)	138	21 (22.5%)	0.007
Current smoker, n (%)	1056	85 (8.0%)	137	14 (10.3%)	0.372
Ethnicity, n (%)					
Indian	1058	514 (48.6%)	138	68 (49.3%)	0.998
Malays		277 (26.2%)		36 (26.1%)	
Chinese		252 (23.8%)		32 (23.2%)	
Others		15 (1.4%)		2 (1.4%)	
Job nature					
Retired, n (%)	1058	580 (54.8%)	138	80 (58.0%)	0.484
Full time, n (%)		223 (21.1%)		24 (17.4%)	0.314
**Self-care, n (%)**					
Self-monitoring of glucose control, n (%)	954	406 (42.6%)	123	68 (33.6%)	0.007
Physical activity (3 times per week or more), n (%)	1046	363 (34.7%)	137	46(33.6%)	0.794
Balanced diet (yes or occasional), n (%)	1038	782 (75.3%)	136	95 (69.9%)	0.167
At least 2 self-care management	1057	498 (47.1%)	138	66 (47.8%)	0.875
**Cardiometabolic risk factors**					
FPG, median (Q1-Q3), mmol/L	1058	7.3 (6.0-9.2)	138	7.2 (6.1-10.2)	0.587
HbA_{1c}, mean ± SD, %	1030	7.7 ± 1.7	136	8.0 ± 2.0	0.057
SBP, mean ± SD, mmHg	1038	135.1 ± 16.5	129	142.1 ± 19.1	<0.001
DBP, mean ± SD, mmHg	1038	80.0 ± 9.8	129	80.6 ± 10.5	0.511
Total Cholesterol, mean ± SD, mmol/L	1029	4.5 ± 1.1	131	4.5 ± 1.3	0.640
LDL-C, mean ± SD, mmol/L	1014	2.5 ± 0.9	130	2.5 ± 1.0	0.865
HDL-C, median (Q1-Q3), mmol/L	1032	1.1 (1.0-1.4)	132	1.1 (0.9-1.4)	0.336
Triglycerides, median (Q1-Q3), mmol/L	1030	1.5 (1.1-2.1)	132	1.6 (1.2-2.1)	0.206
Waist circumference (men), mean ± SD, cm	520	97.2 ± 12.1	84	96.3 ± 11.1	0.531
Waist circumference (women), mean ± SD, cm	519	90.5 ± 10.9	49	89.9 ± 11.6	0.687
BMI, mean ± SD, kg/m^2	1048	27.9 ± 5.1	138	27.4 ± 5.0	0.299
Urinary ACR, median (Q1-Q3), mg/mmol	867	1.8 (0.7-7.0)	123	7.7 (1.6-47.8)	<0.001
eGFR, mean ± SD, mL/min/1.73 m^2	1040	84.1 ± 21.3	137	64.4 ± 24.6	<0.001
Frequency of self-reported hypoglycemia ≥once/month, n (%)	1051	114 (10.8%)	136	19 (14.0%)	0.277
**Comorbidities, n (%)**					
Chronic kidney disease	1058	148 (14.0%)	138	64 (46.4%)	<0.001
Sensory neuropathy	1057	171 (16.2%)	138	25 (18.1%)	0.563
Diabetic retinopathy	1056	51 (4.8%)	138	22 (15.9%)	<0.001
Congestive heart Failure	1058	7 (0.7%)	138	6 (4.3%)	0.002
Cardiovascular disease	1058	208 (19.7%)	138	54 (39.1 %)	<0.001
Ischemic heart disease	1058	25 (2.4%)	138	7 (5.1%)	0.064
Stroke	1058	43 (4.1%)	138	11 (8%)	0.038
Peripheral vascular disease	1058	15 (1.4%)	138	4 (2.9%)	0.263
**Medications, n (%)**					
RASi	1002	604 (60.3%)	134	95 (70.9%)	0.018
Statins	932	780 (83.7%)	118	101 (85.6%)	0.596
Oral glucose-lowering drug	1058	1023 (96.7%)	138	123 (89.1%)	<0.001
Insulin	1058	245 (23.1%)	138	58 (42.0%)	<0.001

Characteristic	n	Alive (n=1058)	n	Deceased (n=138)	p-value
**Treatment targets attainment, n (%)**					
HbA_{1c} <7.0%	1030	422 (41.0%)	136	48 (35.6%)	0.152
BP <130/80 mmHg	1038	229 (22.1%)	129	17 (13.3%)	0.055
LDL-C <2.6 mmol/L	1014	611 (60.3%)	130	84 (64.6%)	0.338
≥2 treatment targets	1014	382 (37.7%)	130	39 (30.0%)	0.088

Footnotes: Data are reported as mean ± standard deviation; n (%), number (percentage); or median (Q1-Q3). Cardiovascular disease included ischemic heart disease, stroke and peripheral vascular disease. ACR, albumin:creatinine ratio; BMI, body mass index; DBP, diastolic blood pressure; eGFR, estimated glomerular filtration rate (in creatinine-based CKD-EPI formula); FPG, fasting plasma glucose; HbA_{1c}, glycated hemoglobin; HDL-C, high-density lipoprotein cholesterol; LDL-C, low-density lipoprotein cholesterol; NGSP, National Glycohemoglobin Standardization Program; RASi, renin-angiotensin-aldosterone system inhibitors; SBP, systolic blood pressure.

S2 Table. Comparison of baseline characteristics between patients who did or did not return for follow-up

Characteristic	n	Returnees (n=826)	n	Non-returnees* (n=232)	p-value
**Assigned Intervention**					
CA-only	826	191 (23.1%)	232	80 (34.5%)	0.648
CA+R		223 (27.0%)		40 (17.2%)	
CA+R+T		412 (49.9%)		112 (48.3%)	
**Sociodemographic**					
Age, years	826	60.0 ± 9.8	232	57.7 ± 12.0	0.008
Duration of diabetes, median (Q1-Q3), years	826	10.0 (5.0-16.0)	228	8.0 (4.0-13.0)	<0.001
Men, n (%)	826	424 (51.3%)	232	105 (45.1%)	0.091
College education or above, n (%)	824	283 (34.3%)	232	75 (32.3%)	0.556
Current smoker, n (%)	825	49 (5.9%)	231	36 (15.6%)	<0.001
Ethnicity, n (%)					
Indian	824	415 (50.2%)	232	99 (42.7%)	0.149
Malays		204 (24.7%)		73 (31.5%)	
Chinese		195 (23.6%)		57 (24.6%)	
Others		12 (1.5%)		3 (1.3%)	
**JADE risk category, n (%)**					
1-2	826	92 (11.1%)	232	21 (9.1%)	0.013
3		579 (70.1%)		184 (79.3%)	
4		155 (18.8%)		27 (11.6%)	
**Self-care, n (%)**					
Self-monitoring of glucose	747	320 (42.8%)	207	87 (42.0%)	0.835
Physical activity (≥3 times/week)	817	292 (35.7%)	229	72 (31.4%)	0.227
Balanced diet	810	613 (75.7%)	229	169 (74.1%)	0.630
At least 2 self-care practices	826	398 (48.2%)	231	100 (43.3%)	0.188
**Cardiometabolic risk factors**					
FPG, median (Q1-Q3), mmol/L	798	7.3 (6.0-9.0)	220	7.6 (6.0-9.8)	0.489
HbA_{1c}, mean ± SD, %	811	7.7 ± 1.7	219	8.0 ± 1.9	0.037
SBP, mean ± SD, mmHg	812	135.0 ± 16.6	226	135.8 ± 16.1	0.516
DBP, mean ± SD, mmHg	812	79.8 ± 9.8	226	80.9 ± 9.6	0.119
Total Cholesterol, mean ± SD, mmol/L	809	4.4 ± 1.0	220	4.7 ± 1.1	0.004
LDL-C, mean ± SD, mmol/L	798	2.5 ± 0.9	216	2.7 ± 0.9	0.002
HDL-C, median (Q1-Q3), mmol/L	811	1.2 (1.0-1.4)	221	1.1 (0.9-1.4)	0.062
Triglycerides, median (Q1-Q3), mmol/L	809	1.5 (1.1-2.0)	222	1.6 (1.1-2.2)	0.044
Waist circumference (men), mean ± SD, cm	415	96.8 ± 12.2	105	98.5 ± 11.8	0.205
Waist circumference (women), mean ± SD, cm	396	90.5 ± 11.0	123	90.8 ± 10.5	0.749
BMI, mean ± SD, kg/m^2	818	27.8 ± 4.9	230	28.5 ± 5.7	0.059
Urinary ACR, median (Q1-Q3), mg/mmol	690	2.0 (0.9-6.5)	176	2.3 (0.8-8.3)	0.073
eGFR, mean ± SD, mL/min/1.73 m^2	817	83.0 ± 21.4	225	87.6 ± 20.8	0.004
Frequency of self-reported hypoglycemia ≥once/month, n (%)	821	92 (11.2%)	230	22 (9.6%)	0.479
**Comorbidities, n (%)**					
Chronic kidney disease	826	123 (14.9%)	232	88 (37.9%)	<0.001
Sensory neuropathy	826	156 (18.9%)	231	16 (6.9%)	0.002
Diabetic retinopathy	826	45 (5.4%)	231	6 (2.6%)	0.076
Congestive heart failure	826	5 (0.6%)	232	2 (0.9%)	0.652
Cardiovascular disease	826	180 (21.8%)	232	29 (12.5%)	0.002
Ischemic heart disease	826	141 (17.1%)	232	22 (9.5%)	0.005
Stroke	826	38 (4.6%)	232	5 (2.2%)	0.130
Peripheral vascular disease	826	13 (1.6%)	232	2 (0.9%)	0.544
Any diabetes-related endpoints	826	474 (57.4%)	232	113 (48.7%)	0.019
**Medications, n (%)**					
RASi	787	485 (61.6%)	215	119 (55.3%)	0.095
Statins	732	620 (84.7%)	200	161 (80.5%)	0.153
Oral glucose-lowering drug	826	799 (96.7%)	232	224 (96.6%)	0.893

Characteristic	n	Returnees (n=826)	n	Non-returnees* (n=232)	p-value
Insulin	826	190 (23.0%)	232	54 (23.3%)	0.930
**Treatment targets attainment, n (%)**					
HbA_{1c} <7.0%	811	339 (41.8%)	219	82 (37.4%)	0.244
BP <130/80 mm Hg	812	183 (22.5%)	226	47 (20.8%)	0.577
LDL-C <2.6 mmol/L	798	496 (62.2%)	216	116 (53.7%)	0.024
≥2 treatment targets	798	313 (39.2%)	216	70 (32.4%)	0.067

*including those who could be contacted and refused to return (n=139) and those who could not be contacted (n=93).

Footnotes: Data were indicated as mean ± standard deviation; n (%), number (percentage) or median (Q1-Q3). Cardiovascular disease includes ischemic heart disease, peripheral vascular disease and stroke. ACR, urine albumin:creatinine ratio; BMI, body mass index; DBP, diastolic blood pressure; eGFR, estimated glomerular filtration rate; FPG, fasting plasma glucose; HbA_{1c}, glycated hemoglobin; HDL-C, high-density lipoprotein cholesterol; LDL-C, low-density lipoprotein cholesterol; RASi, Renin-angiotensin-aldosterone system inhibitors; SBP, systolic blood pressure.

S3 Table. Comparison of clinical characteristics of 826 returnees during the 1-year randomized controlled trial of the JADE Program (baseline and year 1) and post-trial (year 7)

Baseline characteristics	Baseline (n=826)	Year 1 (n=826)	Year 7 (n=826)	p-value
**Sociodemographic**				
Age, years	60.0 ± 9.8	61.2 ± 9.6	67.5 ± 9.8	<0.001
Duration of diabetes, median (Q1-Q3), years	10.0 (5.0-16.0)	11.0 (6.0-17.0)	17.0 (13.0-23.3)	<0.001
Men, n (%)	424 (51.3%)			
College education or above, n (%)	283 (34.3%)	269 (32.6%)	283 (34.3%)	0.001
Current smoker, n (%)	49 (5.9%)	24 (2.9%)	40 (4.8%)	<0.001
**Ethnicity**				
Indian, n (%)	415 (50.2%)			
Malays, n (%)	204 (24.7%)			
Chinese, n (%)	195 (23.6%)			
Others, n (%)	12 (1.5%)			
**Job nature**				
Retired, n (%)	497 (60.2%)	468 (56.7%)	497 (60.2%)	0.100
Full time, n (%)	137 (16.6%)	121 (14.6%)	137 (16.6%)	0.100
Others, n (%)	192 (23.2%)	208 (25.2%)	192 (23.2%)	0.100
**Self-care practices**				
Self-monitoring of blood glucose, n (%)	555 (67.2%)	503 (60.9%)	397 (48.1%)	0.435
Physical activity (3 times per week or more), n (%)	292 (35.4%)	284 (34.3%)	389 (47.1%)	<0.001
Balanced diet (yes or occasional), n (%)	613 (74.2%)	679 (82.2%)	688 (83.3%)	<0.001
**Cardiometabolic risk factors**				
FPG, median (Q1-Q3), mmol/L	7.3 (6.0-9.0)	7.1 (6.1-8.9)	7.1 (5.9-8.7)	0.887
HbA_{1c}, mean ± SD, %	7.6 ± 1.6	7.4 ± 1.5	7.7 ± 1.8	0.312
SBP, mean ± SD, mmHg	135.0 ± 16.6	132.2 ± 14.5	143.5 ± 18.2	<0.001
DBP, mean ± SD, mmHg	79.8 ± 9.8	76.6 ± 8.7	73.7 ± 9.6	<0.001
Total Cholesterol, mean ± SD, mmol/L	4.4 ± 1.0	4.3 ± 0.9	4.3 ± 1.1	<0.001
LDL-C, mean ± SD, mmol/L	2.5 ± 0.9	2.3 ± 0.8	2.3 ± 1.0	<0.001
HDL-C, median (Q1-Q3), mmol/L	1.2 (1.0-1.4)	1.2 (1.0-1.4)	1.2 (1.0-1.5)	<0.001
Triglycerides, median (Q1-Q3), mmol/L	1.4 (1.1-2.0)	1.3 (1.0-1.8)	1.3 (1.0-1.8)	<0.001
Waist circumference (men), mean ± SD, cm	96.8 ± 12.2	98.4 ± 11.4	96.2 ± 11.8	0.024
Waist circumference (women), mean ± SD, cm	90.4 ± 11.0	92.7 ± 10.9	92.1 ± 12.0	<0.001
BMI, mean ± SD, kg/m^2	27.8 ± 4.9	27.7 ± 4.9	27.1 ± 5.3	<0.001
Urinary ACR, median (Q1-Q3), mg/mmol	1.7 (0.7-6.3)	1.7 (0.7-5.8)	4.0 (1.7-14.8)	<0.001
eGFR, mean ± SD, mL/min/1.73 m^2	83.0 ± 22.4	85.2 ± 21.9	78.8 ± 24.8	<0.001
Frequency of self-reported hypoglycemia ≥once/month, n (%)	61 (45.9%)	38 (28.6%)	34 (25.6%)	<0.001
**Comorbidities, n (%)**				
Chronic kidney disease	123 (14.9%)	109 (13.2%)	178 (21.5%)	<0.001
Sensory neuropathy	159 (19.2%)	118 (14.3%)	358 (43.3%)	<0.001
Diabetic retinopathy	47 (5.7%)	53 (6.4%)	95 (11.5%)	<0.001
Congestive heart Failure	5 (0.6%)	5 (0.6%)	20 (2.4%)	<0.001
Cardiovascular disease	180 (21.8%)	175 (21.2%)	310 (37.5%)	<0.001
Ischemic heart disease	141 (17.1%)	141 (17.1%)	226 (27.4%)	<0.001
Stroke	38 (4.6%)	35 (4.2%)	73 (8.8%)	<0.001
Peripheral vascular disease	13 (1.6%)	11 (1.3%)	67 (8.1%)	<0.001
Any diabetes-related complications	474 (57.4%)	335 (40.6%)	571 (69.1%)	<0.001
**Medications, n (%)**				
RASi	485 (61.6%)	492 (59.6%)	589 (71.3%)	<0.001
Statins	620 (84.7%)	612 (74.1%)	638 (77.2%)	<0.001
Oral glucose-lowering drug	799 (96.7%)	743 (90.0%)	776 (93.9%)	0.002
Insulin	190 (23.0%)	176 (21.3%)	334 (40.4%)	<0.001
**Treatment targets attainment, n (%)**				
HbA_{1c} <7.0%	339 (41.0%)	354 (42.9%)	333 (40.3%)	0.519
BP <130/80 mmHg	183 (22.2%)	199 (24.1%)	172 (20.8%)	0.497

Baseline characteristics	Baseline (n=826)	Year 1 (n=826)	Year 7 (n=826)	p-value
LDL-C <2.6mmol/L	496 (60.0%)	532 (64.4%)	569 (69.9%)	<0.001
≥2 treatment targets attained	315 (38.1%)	348 (42.1%)	335 (40.6%)	0.271

Footnotes: Data were indicated as mean ± standard deviation; n (%), number (percentage) or median (Q1-Q3). Cardiovascular disease includes ischemic heart disease, peripheral vascular disease and stroke. ACR, urine albumin:creatinine ratio; BMI, body mass index; DBP, diastolic blood pressure; eGFR, estimated glomerular filtration rate; FPG, fasting plasma glucose; HbA_{1c}, glycated hemoglobin; HDL-C, high-density lipoprotein cholesterol; LDL-C, low-density lipoprotein cholesterol; RASi, Renin-angiotensin-aldosterone system inhibitors; SBP, systolic blood pressure.

S4 Table. Logistic regression analysis of factors associated with any incident diabetes-related endpoints amongst patients without prior history of complications.

	Model 1 (n=573)		Model 2 (n=573)		Model 3 (n=572)		Model 4 (n=572)	
	OR (95% CI)	p-value	OR (95% CI)	p-value	OR (95% CI)	p-value	OR (95% CI)	p-value
**CA+R+T (versus CA only)**	0.94 (0.63-1.39)	0.752	0.94 (0.62-1.43)	0.781	0.95 (0.63-1.44)	0.802	0.87 (0.57-1.34)	0.538
**CA+R (versus CA-only)**	1.41 (0.86-2.30)	0.173	1.30 (0.77-2.18)	0.324	1.29 (0.77-2.17)	0.332	1.28 (0.75-2.17)	0.360
**Age**			1.03 (1.01-1.05)	0.004	1.03 (1.01-1.05)	0.004	**1.03 (1.01-1.05)**	**0.007**
**Men**			1.23 (0.86-1.76)	0.253	1.22 (0.85-1.74)	0.289	1.16 (0.80-1.68)	0.427
**Duration of diabetes**			1.07 (1.04-1.10)	<0.001	1.07 (1.04-1.10)	<0.001	**1.07 (1.04-1.10)**	**<0.001**
**College education or above**					1.04 (0.72-1.52)	0.820	1.04 (0.71-1.52)	0.859
**JADE risk category 3 (vs category 1-2)**							**1.71 (1.04-2.83)**	**0.035**
**JADE risk category 4 (vs category 1-2)**							**35.85 (4.64-277.24)**	**<0.001**

Footnotes: OR, odds ratio (95% confidence intervals).
